# Associations of the related factors of depression, social support and social participation in *kyungro‐dang* among older adults in South Korea: A structural equation modelling analysis

**DOI:** 10.1002/nop2.661

**Published:** 2020-10-19

**Authors:** Hocheol Lee, Seokjun Moon, Geurum Song, Eun Woo Nam

**Affiliations:** ^1^ Department of Health Administration Yonsei University Graduate School Wonju Korea; ^2^ Public Health Network and Quality Enhancement Team Center for Public Healthcare National Medical Center Seoul Korea; ^3^ Department of Health Administration College of Health Science Yonsei University Wonju Korea

**Keywords:** community care, community‐based long‐term care facilities, depression, nurses, nursing, older people

## Abstract

**Aim:**

This study aimed to investigate the correlations between depression, social support and social participation among older adults in farming villages and identify their associations with the use of senior leisure and welfare facilities, called *kyungro‐dang*, so as to provide foundational data for promoting community care in rural farming communities.

**Design:**

A cross‐sectional design was used to identify kyungro‐dang use among the 156 participants—older adults aged 65.

**Methods:**

Data were analysed using descriptive statistics, Pearson's correlation analysis, factor analysis and structural equation modelling.

**Results:**

Older adults with high social support and social participation tended to use *kyungro‐dang* less. Our findings contribute foundational data to promote community care through *kyungro‐dang*. Ministry of Health and Welfare (MoHW) plans to enforce to “Comprehensive community care plan” through *kyungro‐dang* in rural area. The result of this study shows the relation between *Kyungro‐dang* and social support, social participation, depression. This study recommends that MoHW in Korea focuses on enhance social support and social participation to gather before policy implications.

## 1 INTRODUCTION

South Korea became an ageing society in 2000, with the proportion of individuals aged 65 years and older exceeding 7% of the entire population. Then, in 2018, with the proportion of older adults constituting 14.3% of the entire population, the country attained the status of an aged society. By 2026, when older adults will account for 20% of the total population, South Korea is projected to become a super‐aged society (OECD, 2018). Subsequently, by 2050, the proportion of older adults is predicted to exceed 41.0% of the total population, making the country the fifth oldest in the world (United Nation, 2017).

Such changes in the population structure have led to various social and economic problems. One of the greatest social problems is the ever‐increasing need for social care owing to the changes in the types of households composed of older adults (England, 2016). According to the National Survey of Older Adults (Ministry of Health & Welfare, 2017), the proportion of older adults living with their children was the highest in 2004 (38.6%) and the lowest in 2017 (23.7%). In other words, the proportion of children supporting their older adult parents is declining and older adult couples living by themselves are becoming a burden for each other (England, 2016). Thus, the provision of social care for this expanding population is a serious concern.

Economically, the cost of care shouldered by the National Health Insurance Service in Korea for the older adult population has increased; with the society's rapid ageing, health insurance medical expenses for the population aged 65 and older jumped from 2.3 trillion KRW (17.5%) in 2000 to 27.7 trillion KRW (39.9%) in 2017 (Oliveira Martins & de la Maisonneuve, 2014). According to the National Health Insurance Service (Shin et al., 2012), medical costs for older adults are projected to account for more than half of the total health insurance expenditure in 2030, reaching 91.3 trillion KRW. The major causes of high medical costs for older adults are “social admission” to hospital, attributed to the absence of caregivers and poor living conditions and the “revolving door phenomenon” in the medical and welfare system (Botha et al., 2010).

To address such social and economic problems, the Ministry of Health and Welfare (MoHW) has announced the implementation of the “comprehensive community care plan” to provide services, such as care, nursing and protection, mainly for community‐dwelling older adults (Ministry of Health & Welfare, 2018). Through this plan, the MoHW aims to enhance residential, medical, nursing and care services to ensure that older adults can enjoy a healthy older adulthood at their places of living. According to the National Survey of Older Adults (Ministry of Health & Welfare, 2017), 57.6% of older adults wish to live out their remaining years where they have been living even if they experience movement discomfort.

The MoHW plans to enforce the comprehensive community care plan through *kyungro‐dang*—senior leisure and welfare facilities like senior centres. Per South Korea's Welfare of Older Persons Act, *kyungro‐dang* provide a place and programmes for older adults in the community to interact with one another, engage in hobbies, participate in workshops, exchange information and enjoy other leisure activities; older adults aged at least 65 years are the eligible users of these facilities. To promote community‐based prevention and management of chronic diseases in older adults, the MoHW plans to launch and promote health prevention and management programmes and add 48,000 more *kyungro‐dang* by 2025 (Chun et al., 2009). Further, through the comprehensive community care plan announced in 2019, the MoHW specified its intention to transform *kyungro‐dang* into spaces that promote older adults’ social participation and adjustment to ageing beyond their current use as spaces for gatherings, social interaction, rest and activities (Ministry of Health & Welfare, 2018).

## 2 BACKGROUND

The literature provides evidence of the benefits of putting *kyungro‐dang* to such uses (Ministry of Health & Welfare, 2018). In one study (Turner, 2004), older adults using *kyungro‐dang* displayed higher rates of successful ageing, perceived health status, satisfaction with life and satisfaction with their relationship with children, as well as experiencing lower depression, than those who did not use *kyungro‐dang*. Furthermore, the incidence of disease and risk factors has been reported to be about 71.3% lower among older adults who use *kyungro‐dang* than those who use other types of senior leisure and welfare facilities (Lee & Song, 2015).

The World Health Organization (WHO) emphasizes the importance of maintaining an age‐appropriate level of involvement in life in older adulthood, defined as active ageing (World Health Organization, 2002). It has been suggested that utilizing *kyungro‐dang* is associated with active ageing (Turner, 2004). According to a previous study (Chung et al., 2011), older adults who use *kyungro‐dang* have higher life satisfaction and experience more positive ageing, such as improved physical functioning and psychological stability, than do those who do not use *kyungro‐dang*. Active ageing is reflected in various aspects of life in older adulthood, including psychological characteristics, health status, social relationships, leisure activities, depression and social support (Kim et al., 2011; Mollenkopf & Walker, 2007). Older adults with high social participation have been found to use *kyungro‐dang* more often than those with low social participation (Park et al., 2012). In particular, older adults’ social participation is reportedly influenced by depression and social support. Older adults with higher social support demonstrate more social participation and use *kyungro‐dang* more often; studies have confirmed that older adults who have high support from friends and the family they live with use *kyungro‐dang* more often, which, in turn, has a positive impact on their satisfaction with life (Chen, 2001). Moreover, social support relieves daily stress and brings about mental stability in older adults, which lowers depression (Cassel, 1976; Gana et al., 2013). The UK government implement the policies for the depression of older people, by appointing a Ministry for loneliness. As a result, social support through the older people network was effective in reducing depression.

The present study aimed to identify the correlations between depression, social support and social participation and examine their associations with *kyungro‐dang* use among older adults living in a farming village. Ultimately, we aimed to provide foundational data to promote community care. The specific objectives are as follows:

- To identify the associations between depression, social support and social participation based on their correlations in a sample of older adults living in a rural area.
- To examine the associations of depression, social support and social participation with *kyungro‐dang* use and subsequently identify the direct, indirect and total associations among older adults living in a rural area.

## 3 METHODS

### 3.1 Design

**Figure 1:**
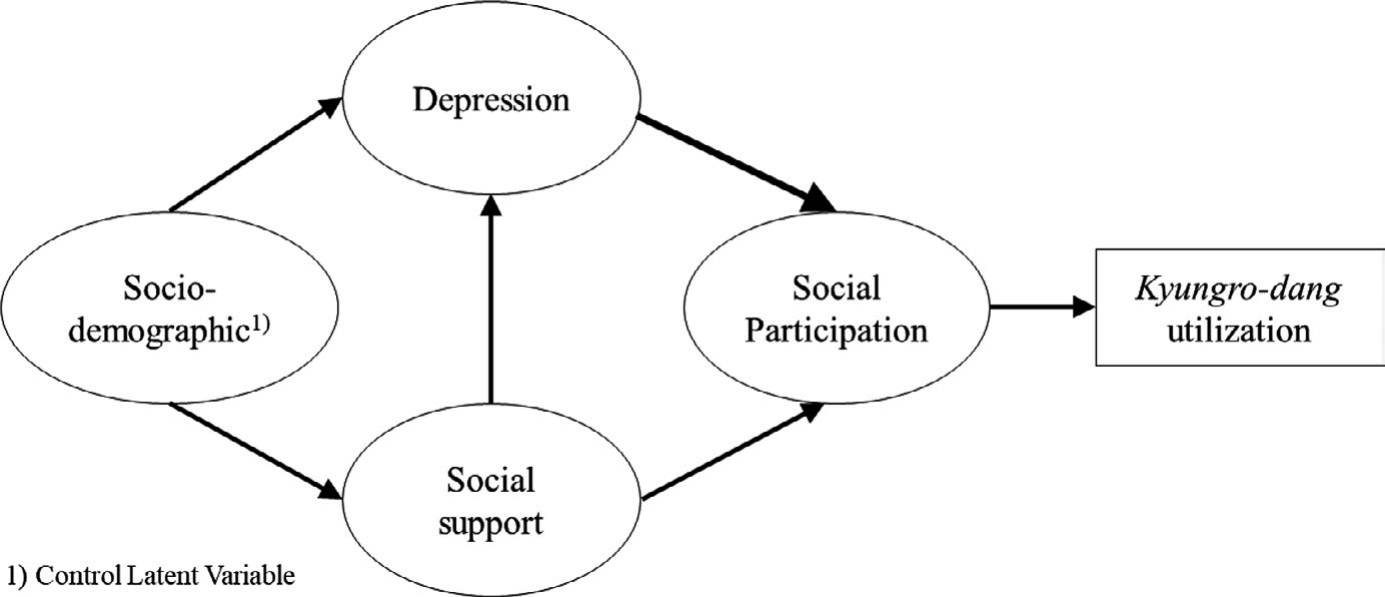
The research model

This study employed a cross‐sectional design to investigate the correlations among depression, social support and social participation and then identify their effects on the use of *kyungro‐dang* among older adults aged 65 years and older living in a rural area, namely a farming village (Figure 1).

### 3.2 Participants

Older adults aged 65 years and older who lived in H‐*myeon*, W City, were enrolled in this study. The sample size was computed using G*Power 3.1. With an effect size of 0.1, power of 0.95 and significance level of 0.05, the minimum sample size was calculated to be more than 160. Potential participants were selected via proportionate probability sampling from four *ri* units, and 181 older adults were surveyed. After excluding 25 respondents who dropped out during the survey or provided insincere or missing responses, data from 156 participants were analysed.

### 3.3 Data collection

Data were collected via face‐to‐face interviews using a structured questionnaire from May 13–24, 2019. Face‐to‐face interviews were conducted with older adults who were able to communicate; those experiencing communication difficulties as a result of physical or mental diseases were excluded. The surveys were conducted through home visits with those on the list of households with older adults obtained with the permission of the *myeon* office worker in charge of *kyungro‐dang* and the head of the village. A trained survey administrator visited older adults in the target region and conducted the surveys.

### 3.4 Measurement and variables

#### 3.4.1 Sociodemographic characteristics

The sociodemographic characteristics of interest included participants’ sex, age, length of residence in the village, type of household, marital status, educational level, religion and monthly income. Household type was classified as “living with a spouse only,” “living alone” or “other.” Marital status was classified as “married,” “widowed,” “divorced” or “separated.” Educational level was classified as “no education,” “elementary school,” “middle school,” “high school” or “college or higher.” Religion was divided into “yes” and “no,” and monthly income was divided into “<500,000 KRW,” “500,000–1,000,000 KRW” and “>1,000,000.”

#### 3.4.2 Social support

Social support was measured using the Multidimensional Scale of Perceived Social Support (Zimet et al., 1988) an eight‐item measure consisting of subscales for friends and significant others. Each item is rated on a five‐point Likert scale from *strongly disagree* (1) ‐ *strongly agree* (5).

#### 3.4.3 Depression

Depression in older adults is often assessed with the 15‐item Geriatric Depression Scale (GDS) developed in 1986 (D'Ath et al., 1994). In this study, we used a Korean version of the scale adapted in 1996 (Park et al., 2006). The highest score for the GDS is 15, and a score of 6 or higher indicates depression symptoms.

#### 3.4.4 Social participation

Social participation was measured using a Korean version of the Provision of Social Relationships scale (Cutrona & Russell, 1987; Hyun Cha et al., 2012). The tool consists of 10 items: six for satisfaction derived from new expectations and sense of agreement gained in social gatherings and four for degree of desire for social participation. Each item is rated on a five‐point Likert scale from *strongly disagree* (1) to *strongly agree* (5), with higher scores indicating higher social participation.

#### 3.4.5 Kyungro‐dang use

*Kyungro‐dang* utilization was measured based on the response (*yes* = 1 point or *no* = 0 points) to the question, “Have you used *kyungro‐dang* in the past 6 months?”

### 3.5 Data analysis

We performed SEM on SPSS 25.0 and AMOS 21 (IBM Corp.) to investigate the structural relations of social support and depression with social participation, as well as the direct and indirect associations of these structural relations with *kyungro‐dang* use. Analyses were performed as follows.

First, the general characteristics of the observed variables, which were used to measure the latent variables of depression, social support and social participation, were examined with frequency analysis. Second, the correlations among latent variables were analysed with Pearson's correlation coefficients. Third, factors for latent variables were analysed with exploratory factor analysis (EFA) and factors were extracted with principal component analysis (PCA) on a correlation matrix. For factor rotation, varimax rotation was performed. The validity of the factor analysis was tested with Bartlett's test of sphericity and the Kaiser‐Meyer‐Olkin (KMO) test. Fourth, SEM analysis was performed to examine the relations among the latent variables that affected *kyungro‐dang* use. Spearman's correlation coefficient matrix was used for input, and parameter estimation was performed with the maximum likelihood method. Fifth, the goodness of fit test was performed for the SEM. Goodness of fit was analysed with the goodness of fit index (GFI), adjusted goodness of fit index (AGFI), root mean square residual and comparative fit index (CFI). The modification index was used to enhance the goodness of fit of the model. Finally, the associations of the independent and mediating variables with the dependent variable were examined by analysing the direct, indirect and total associations of the latent variables through SEM.

## 4 RESULTS

### 4.1 Participant characteristics

Of the 156 participants analysed, 46 (29.5%) were men and 110 (70.5%) were women. The mean age was 78.3 ± 6.6 years, and the mean length of residence in the village was 31.6 ± 26.0 years. Regarding household type, 54 (34.6%) lived with only their spouse, 70 (46.2%) lived alone and 30 (19.2%) were categorized as “other.” Regarding educational level, 64 participants (41.0%) were uneducated, 52 (33.3%) were elementary school graduates, 17 (10.9%) were middle school graduates, 17 (10.9%) were high school graduates and 6 (3.8%) were college graduates or higher. Meanwhile, 85 (54.5%) professed a religion. Regarding monthly income, 90 (57.5%) earned below 500,000 KRW and 44 (28.2%) earned between 500,000 and 1,000,000 KRW (Table 1).

**Table 1:** General characteristics of the participants (*N* = 156)

Variables	Category	*N* (%)	Mean ± *SD*
Sex	Men	46 (29.5)	
	Women	110 (70.5)	
Age (years)	65–69	15 (9.6)	78.37 ± 6.64
	70–74	31 (19.9)	
	75–79	35 (22.4)	
	80–84	44 (28.2)	
	≥85	31 (19.9)	
Residence period (years)	≤5 years	32 (20.5)	31.66 ± 26.08
	5–9 years	11 (7.1)	
	10–14 years	11 (7.1)	
	15–19 years	8 (5.1)	
	≥20 years	93 (59.6)	
Household type	Living with spouse	54 (34.6)	
	Living alone	70 (46.2)	
	Other	30 (19.2)	
Educational level	Uneducated	64 (41.0)	
	Elementary graduates	52 (33.3)	
	Middle school graduates	17 (10.9)	
	High school graduates	17 (10.9)	
	College graduates or higher	6 (3.8)	
Religion	No	71 (45.5)	
	Yes	85 (54.5)	
Monthly Income	≤500,000 KRW	90 (57.7)	
	500,000–1,000,000 KRW	44 (28.2)	
	≤1,000,000 KRW	22 (14.1)	
Depression (score of 1–5)			1.67 ± 1.39
Social support (score of 1–5)			3.03 ± 1.17
Social participation (score of 1–5)			3.58 ± 1.13

### 4.2 Correlation coefficients between depression, social support and social participation

**Figure 2:**
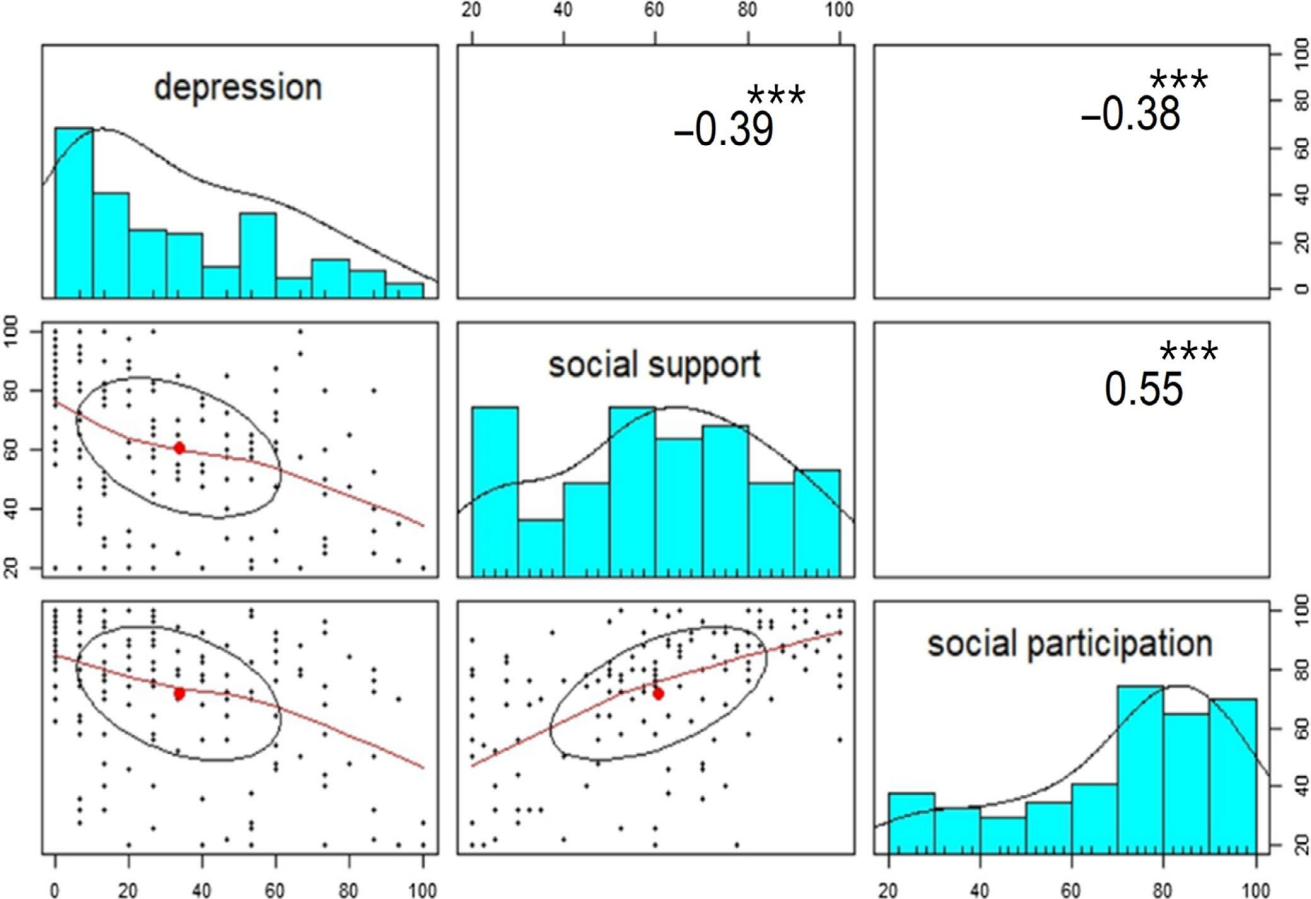
Correlation coefficients among depression, social support and social participation

The correlations between depression, social support and social participation were analysed with Pearson's correlation analysis (Figure 2). Depression was negatively correlated with social support (*γ* = −0.39, *p* < .001^***^) and social participation (*γ* = −0.38, *p* < .001^***^). Social support was positively correlated with social participation (*γ* = 0.55, *p* < .001^***^).

### 4.3 Reliability and validity of EFA

Principal component analysis identified three components for depression, two for social support and two for social participation. The Cronbach's alpha values for the latent variables were all above 0.7:0.797 for depression, 0.896 for social support and 0.941 for social participation. The KMO values were all above 0.8:0.868 for depression, 0.850 for social support and 0.941 for social participation. Depression, social support and social participation were all statistically significant, as demonstrated by Bartlett's test of sphericity (*p* < .001^***^).

### 4.4 Goodness of fit of the research model

Table 2 depicts the goodness of fit of the SEM as analysed with control, observed and latent variables. For absolute fit indices, GFI was 0.932, confirming that the model had a more than acceptable fit. RMSEA was 0.056, or below the 0.1 standard (United Nations Economic Commission for Europe, 2017), thus confirming a good fit. For normed fit indices, both TLI and CFI values were above 0.90, confirming a better fit than the independent model. NFI was below 0.90, whereas the normed fit indices were acceptable. AGFI, a parsimonious fit index, was 0.886, confirming a good fit.

**Table 2:** Goodness of fit of the research model (*N* = 156)

	Absolute			Incremental			Parsimonious
χ^2^ (*p*‐value)	GFI	RMR	RMSEA	TLI	NFI	CFI	AGFI
120.85 (*p* < .001)	0.932	0.168	0.056	0.917	0.846	0.941	0.886

Abbreviations: AGFI, adjusted goodness of fit index; CFI, comparative fit index; GFI, goodness of fit index; NFI, normed fit index; RMR, root mean square residual; RMSEA, root mean square error of approximation; TLI, Tucker‐Lewis index.

### 4.5 SEM

The coefficients for sociodemographic characteristics, depression, social support, social participation and *kyungro‐dang* use were analysed. Social support had a significant negative association with depression (*p* < .001^***^), with a regression coefficient of −0.121 and a significant positive association with social participation (*p* = .006^**^), with a regression coefficient (*β*) of 0.369. Social participation had a significant negative association with *kyungro‐dang* use (*p* < .001^***^), with a regression coefficient of −0.555 (Table 3).

**Table 3:** Path estimates for the research model (*N* = 156)

	Path	Regression weight	Standardized regression weight	*SE*	CR	*p*
1	Sociodemographic characteristics → Social support	0.118	0.095	0.138	0.860	.390
2	Sociodemographic characteristics → Depression	−0.004	−0.012	0.038	−0.111	.912
3	Social support → Depression	−0.121	−0.437	0.034	−3.539	<.001***
4	Depression → Social participation	−0.400	−0.157	0.271	−1.476	.140
5	Social support → Social participation	0.369	0.522	0.136	2.723	.006**
6	Social participation → *Kyungro‐dang* use	−0.550	−0.525	0.148	−3.723	<.001***

Abbreviations: CR, critical ratio; *SE*, standard error.

* *p* < .05.

** *p* < .01.

*** *p* < .001.

The total associations of the latent variables of depression, social support and social participation among older adults with *kyungro‐dang* use were examined based on the direct and indirect correlations (Table 4). Social support had a significant negative indirect association with *kyungro‐dang* use (−0.230, *p* = .025^*^), whereas social participation had a significant negative association (−0.550, *p* = .025^*^).

**Table 4:** Direct, indirect and total associations (*N* = 156)

	Path	Direct effect	*p*	Indirect effect	*p*	Total effect	*p*
7	Social support → *Kyungro‐dang* use	−	−	−0.230	.025*	−0.230	.025*
8	Depression → *Kyungro‐dang* use	−	−	0.220	.163	−0.400	.327
9	Social participation → *Kyungro‐dang* use	−0.550	.025*	−	−	−0.550	.025*

* *p* < .05.

** *p* < .01.

*** *p* < .001.

## 5 DISCUSSION

This study identified the associations between depression, social support and social participation among older adults living in a rural area using SEM, subsequently examining their correlations with *kyungro‐dang* use. Ultimately, this study aimed to provide foundational data for improving *kyungro‐dang* use, which is important for the promotion of community care.

Regarding the participants’ sociodemographic characteristics, the sex distribution (29.5% men, 70.5% women) differed from that in Gangwon‐do (43.7% men, 56.3% women) as reported by the 2017 National Survey of Older Adults and the W City Annual Statistics Report (41.0% men, 59.0% women) (Ministry of Health & Welfare, 2017). Moreover, there are more women than men in farming villages (Ministry of Health & Welfare, 2017). However, during this survey, which was conducted in May, it was observed that men tended to spend more time in the fields than women, despite it being relatively less busy than the farming season in August; this tendency made it difficult to maintain a balanced sex ratio.

The greatest number of our participants belonged to the 80–84 age group (28.2%), whereas the ages of 65–69 (28.5%) were the most common in Gangwon‐do overall. The difference is because we only included *eup*, *myeon* and *ri* units in our study and excluded cities. The greatest number of participants tended to be uneducated (41.0%), followed by elementary graduates (33.3%). According to the 2017 National Survey of Older Adults (Ministry of Health & Welfare, 2017), 40.3% of older adults in Gangwon‐do are elementary graduates and 25.8% are uneducated. A previous study has found that a higher percentage of older adults in farming villages are uneducated than in urban regions (Drop, 2018).

This study identified the associations between depression, social support and social participation through correlational analysis. Pearson's correlation analysis revealed a statistically significant negative correlation between depression and social support. A study of older adults aged 65 years and older using the same tool as that used in this study reported a similar correlation between depression and social support (Turner, 2004). In another study using a different tool, social support was classified into emotional, informational, material and evaluational support, all of which had a negative association with depression, in the order of emotional, informational, evaluational and material support (Cassel, 1976). Many other studies have confirmed that depression significantly decreases with increasing social support, which was also confirmed in our study (Gana et al., 2013; Gureje et al., 2007). Depression in older adults had a statistically significant negative correlation with social participation, which has been found in previous studies as well (Oddone et al., 2011).

According to the National Survey of Older Adults (Ministry of Health & Welfare, 2017), about 11.3% of older adults in Gangwon‐do experienced depression symptoms and the depression score measured with the same tool used in this study was 2.8 out of 5. The depression score in our participants was 1.6, which was comparatively lower. As depression in older adults in farming communities is statistically related to social support and social participation, additional studies are needed of the latent variables associated with depression in rural areas, particularly farming regions.

This study investigated the associations of depression, social support and social participation with the *kyungro‐dang* use among older adults in farming communities, subsequently examining the direct, indirect and total associations among latent variables. The path coefficient for the correlations of social support with depression was significant in SEM, and the results of the correlation analysis were similar to those of previous studies.

In our SEM, the regression coefficient for the total association of social participation with *kyungro‐dang* use was significant. In other words, older adults with higher social participation scores used *kyungro‐dang* less. This contradicts previous findings that older adults who use *kyungro‐dang* have higher social participation scores (Turner, 2004). The difference may be attributable to the fact that whereas the previous study (Turner, 2004) involved only older adults who use *kyungro‐dang*, our study included users and non‐users of *kyungro‐dang*. Further, based on a previous finding (Park et al., 2012) that older adults who actively engage in socioeconomic activities, religious activities and clubs, as well as attending other senior welfare facilities and schools for older adults, have higher social participation scores, we can infer that older adults with high social participation use *kyungro‐dang* less frequently owing to their engagement in other activities. There was a contrasting finding that *kyungro‐dang* use is 2.399 times higher in those with a stronger social network. However, in another report (Park et al., 2015), older adults with higher incomes reported utilizing *kyungro‐dang* less frequently and showed higher social participation; these results are similar to ours in suggesting that older adults with higher social participation tend not to use *kyungro‐dang*.

SEM confirmed that social support is correlated with *kyungro‐dang* use, with a significant total association coefficient of −0.230 (*p* = .025). In other words, older adults with higher social support use *kyungro‐dang* less frequently. This contradicts previous findings that older adults with higher instrumental and emotional support from their families use *kyungro‐dang* more frequently (Chen, 2001; Park et al., 2012). We can infer that older adults with higher trust and support from family and friends use *kyungro‐dang* less frequently because they spend more time with their family and friends. Further, it has been reported that older adults living in farming villages with more household income have higher social support from family and friends and these people do not use *kyungro‐dang* (Ministry of Health & Welfare, 2017). In light of these findings, older adults with higher social support are likely not to use *kyungro‐dang*, as found in our study.

The government announced the measure to promote the use of *kyungro‐dang* for health prevention and management programmes in communities through the comprehensive community care plan as integrated care (Ministry of Health & Welfare, 2018). The community care plan in Korea is based on the concept of integrated care, which is a type of comprehensive care encompassing various fields, indicating that medical health care, nursing care, residential facilities and social welfare facilities are socially combined (Ministry of Health & Welfare, 2018).

The WHO Regional Office in Europe classified the integration scale as: (a) Individual models of integrated care, (b) Group and disease specific models and (c) Population‐based models (WHO Regional Office for Europe, 2016). The group and disease specific models encourage participation of older adults through community programmes (WHO Regional Office for Europe, 2016). In this study, it was found that older adults with low social support and social participation used *kyungro‐dang* significantly more. In this regard, the selection of older adults whose level of social support and social participation is low, which indicates higher opportunity of being depressed, is considered more effective. This way, they can become the recipients of community care for integrated care in Korea and reduce depression through community programmes in *kyungro‐dang*, which is likely to be visited by older adults.

A depression programme was implemented at a senior centre in the United States as part of the group models of integrated care. Of the 217 programme participants, 88% said they visited the senior centre for friendship and social support. As a result, 94% of the participants made friends in the senior centre and 4.5% of them reported improvement in depression. This can be used as an exemplary case for policymaking on community care for integrated care in Korea (Fulbright, 2010).

In addition, a study on senior centre programmes conducted in Seattle and Tennessee in the USA found that 114 out of 140 study patients from four Seattle senior centres showed improvements in depression (Raue et al., 2019). In addition, physical and mental health improved in 385 patients in the Tennessee senior centre programme (Aday et al., 2019).

Canada has implemented an integrated care programme in its senior centre as part of its social prescription, which has helped improve depression, anxiety, emotional disorder, physical functioning and social participation among older adults who participated in the programme (Novek et al., 2013). This observation corresponds with the findings of this study that depression in older adults with high social support and social participation is significantly lowered. In other words, older adults who have low social support and social participation are more likely to have depression and use *kyungro‐dang* significantly more. Therefore, it is reasonable for the Korean community care programme to be conducted in *kyungro‐dang*.

In the UK, social participation opportunities and social support to the older adults are provided through social prescription via various programmes such as art therapy and horticultural therapy. As a result, depression in older adults has decreased and voluntary and active attitudes have improved (Vogelpoel & Jarrold, 2014).

Korea's community care for integrated care policy was planned focusing on the older adult population in the community; pilot operations in this regard are being conducted around the community. Particularly, in rural areas, *kyungro‐dang* is the only facility where older adults can have opportunities for social participation, unlike in urban areas (Turner, 2004). As has been reported in this study and based on the fact that older adults with low social support and social participation use *kyungro‐dang* significantly more, a detailed study on the measures to introduce the integrated care programme in *kyungro‐dang* is warranted. Furthermore, with *kyungro‐dang* as the centre of the community care for integrated care network, the opportunity of effective linkage with primary healthcare institutions, community centres and local health clinics needs to be discussed in further studies. In addition, further research and discussion is required to ensure that *kyungro‐dang*/senior centres, which are tailored for the super‐aged society of Korea, can be developed based on overseas cases such as the social prescription programme of the UK, the senior centre programme of the US, the social prescription senior centres of Canada and community general support centres of Japan.

### 5.1 Limitations

This study had some limitations. First, ratio of women to men was not balanced among participants in this study. This limitation has been documented in a previous study of older adults in rural areas (United Nations Economic Commission for Europe, 2017). Furthermore, the proportion of women compared with men increases with age (OECD, 2018), and considering that the ratio of women to men among older adults aged 80 years and older in myeon units is about 7:3 (Cutrona & Russell, 1987), the sex ratio in our participants was similar. Second, the population among older adults in the research area was about 1,513 people. Therefore, it is difficult to make generalizations about the national level in Korea from the finding. In future studies, using national data by scaling up could be given priority.

## 6 CONCLUSIONS

The results showed that social support and social participation have a negative correlation with *kyungro‐dang* use among older adults in farming villages. In other words, in contrast to previous findings that older adults with higher social support and social participation use *kyungro‐dang* more often, our findings suggest that older adults in farming communities with high social support and social participation use *kyungro‐dang* less. To promote the use of *kyungro‐dang* as one of the major platforms of the plan to provide older adults with comprehensive community and nursing care, in‐depth studies on how factors such as social support and social participation influence *kyungro‐dang* use are needed. Empirical findings are needed to serve as the bases for the successful promotion of community care.

## CONFLICT OF INTEREST

The authors have no competing interests to declare.

## AUTHOR CONTRIBUTIONS

HCL and EWN: Study design. HCL, SJM and GRS: Data collection. HCL: Data analysis. HCL, SJM, GRS and EWN: Data interpretation. All authors: Contribution of the first draft. EWN: Final version approval.

## ETHICAL APPROVAL

This study was approved by the concerned institutional review board (no. 1041849‐201908‐SB‐118‐01). Informed consent was obtained from each respondent prior to data collection.

## Data Availability

The original data are in Korean language. Due to the promise of confidential presentation of the participants, we prefer not to share these data.
